# Ring stage classification of *Babesia microti* and *Plasmodium falciparum* using optical diffraction 3D tomographic technique

**DOI:** 10.1186/s13071-022-05569-0

**Published:** 2022-11-17

**Authors:** Ernest Mazigo, Hojong Jun, Jeonghun Oh, Wasiq Malik, Johnsy Mary Louis, Tong-Soo Kim, Se Jin Lee, Sunghun Na, Wanjoo Chun, Won Sun Park, Yong-Keun Park, Eun-Taek Han, Min-Jae Kim, Jin-Hee Han

**Affiliations:** 1 Department of Medical Environmental Biology and Tropical Medicine, Kangwon National University School of Medicine, Chuncheon, Republic of Korea; 2 Department of Tropical Medicine, Inha University College of Medicine, Incheon, Republic of Korea; 3 Department of Physics, Korea Advanced Institute of Science and Technology (KAIST), Daejeon, Republic of Korea; 4 Korea Advanced Institute of Science and Technology (KAIST) Institute for Health Science and Technology, KAIST, Daejeon, Republic of Korea; 5 Department of Obstetrics and Gynecology, Kangwon National University Hospital, Kangwon National University School of Medicine, Chuncheon, South Korea; 6 Department of Pharmacology, Kangwon National University School of Medicine, Chuncheon, Republic of Korea; 7 Department of Physiology, Kangwon National University School of Medicine, Chuncheon, Republic of Korea; 8 Tomocube Inc., Daejeon, Republic of Korea; 9 Department of Infectious Diseases, Asan Medical Center, University of Ulsan College of Medicine, Seoul, Republic of Korea

**Keywords:** Red blood cells, Optical diffraction tomography, *Plasmodium falciparum*, *Babesia microti*, Three-dimensional refractive index

## Abstract

**Background:**

*Babesia* is an intraerythrocytic parasite often misdiagnosed as a malaria parasite, leading to inappropriate treatment of the disease especially in co-endemic areas. In recent years, optical diffraction tomography (ODT) has shown great potential in the field of pathogen detection by quantification of three-dimensional (3D) imaging tomograms. The 3D imaging of biological cells is crucial to investigate and provide valuable information about the mechanisms behind the pathophysiology of cells and tissues.

**Methods:**

The early ring stage of *P. falciparum* were obtained from stored stock of infected RBCs and of *B. microti* were obtained from infected patients during diagnosis. The ODT technique was applied to analyze and characterize detailed differences between *P. falciparum* and *B. microti* ring stage at the single cell level. Based on 3D quantitative information, accurate measurement was performed of morphological, biochemical, and biophysical parameters.

**Results:**

Accurate measurements of morphological parameters indicated that the host cell surface area at the ring stage in *B. microti* was significantly smaller (140.2 ± 17.1 µm^2^) than that in *P. falciparum* (159.0 ± 15.2 µm^2^), and sphericities showed higher levels in *B. microti*-parasitized cells (0.66 ± 0.05) than in *P. falciparum* (0.60 ± 0.04). Based on biochemical parameters, host cell hemoglobin level was significantly higher and membrane fluctuations were respectively more active in *P. falciparum*-infected cells (30.25 ± 2.96 pg; 141.3 ± 24.68 nm) than in *B. microti* (27.28 ± 3.52 pg; 110.1 ± 38.83 nm). The result indicates that *P. falciparum* more actively altered host RBCs than *B. microti*.

**Conclusion:**

Although *P. falciparum* and *B. microti* often show confusable characteristics under the microscope, and the actual three-dimensional properties are different. These differences could be used in differential clinical diagnosis of erythrocytes infected with *B. microti* and *P. falciparum*.

**Graphical Abstract:**

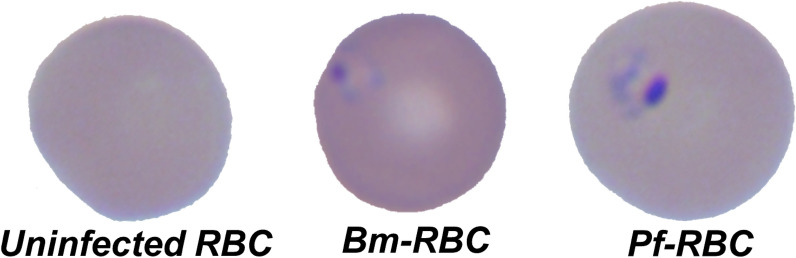

## Background

The Apicomplexa phylum is known for having important obligate intracellular parasites carrying human infectious diseases and has been responsible for major social and economic burdens [1, 2]. Apart from phylogeny-based studies showing common ancestry [3], comparative genomics show that Apicomplexa parasites share some commonalities in invasion of host cells and genetic level metabolism [4]. Sometimes, it has been very difficult to differentiate host invasion characteristics of Apicomplexa members. However, for effective control and management of human invasive parasites, the pathophysiological mechanisms and ecological dynamics between host cells and parasites must be clearly known.

Babesiosis and malaria are intraerythrocytic protozoan parasites of genera *Babesia* and *Plasmodium* from Apicomplexa phylum transmitted by tick and female *Anopheles* mosquitoes, respectively [5, 6]. Over 1 million deaths reported annually were caused by hematotropic parasites of the genus *Babesia* [2] and *Plasmodium* species, contributing > 10% of all global deaths [5, 7]. There are several species of *Babesia* but the parasitic species most infective to humans is *Babesia microti* [8, 9]. So far, co-infection of *B. microti* and *Plasmodium* parasites has also been reported in several areas where the two diseases are endemic [8–10].

Both *Babesia* and *Plasmodium* replicate in and eventually destroy erythrocytes of their mammalian hosts and are known to cause severe fetal diseases in humans [11, 12]. Unlike *Plasmodium* species, intraerythrocytic cycles of *Babesia*, including their invasion, growth, division, and egress, are still not fully understood. In addition, development stages of *Babesia* present similar morphological features to those of *Plasmodium falciparum* [7, 8], and *Babesia* patients usually present with malaise, fever, fatigue, and headache, sometimes accompanied by hemolytic anemia and thrombocytopenia similar to malaria symptoms [13]. Such similarities have been a challenge in clinical diagnosis and management of the two diseases.

Microscopy with Giemsa stain blood smear is the primary technique for diagnosis of both *B. microti*-infected red blood cells (*Bm*-RBCs) and *P. falciparum*-infected red blood cells (*Pf*-RBCs) [6, 14]. However, the approach has several limitations especially in discriminating *Bm*-RBCs and *Pf*-RBCs. The technique largely depends on the experiences of professional technicians. In some areas, microscopy technique has been reported to lead to misdiagnosis between babesiosis and malaria and thus inappropriate management [15]. Electron microscopy has been optional when diagnosing the two parasitic infections (*Pf*-RBCs and *Bm*-RBCs) [16, 17]. Although the electronic microscope provides high-resolution imaging, it cannot be used for imaging cell dynamics and intact cell information as it requires a metal coating that can damage parasite membranes [17, 18].

This study describes the use of tomographic techniques to differentiate 3D morphological structures of *Bm*- and *Pf*-RBCs. We assessed different parameters of *B. microti*- and *P. falciparum*-infected RBCs using optical diffraction tomography (ODT). We used the early ring stage 3D structures of human RBCs that were clinically parasitized. Making the clinical diagnosis of the two diseases according to the ring stage is confusing [19]. Therefore, our study considered ODT as a unique quantitative phase imaging (QPI) technique to present experimental results on *Bm*- and *Pf*-RBCs. This study presents differences in 3D refractive index (RI) tomographs between *Pf*- and *Bm*-RBCs based on morphological, biochemical, and biophysical parameters. The internal detailed structures of live host cells and invaded microbes were also investigated without any exogenous labeling agents such as fluorescent dye. The result highlights detailed differences between *B. microti* and *P. falciparum* at the ring stage which should facilitate field diagnosis. This technique provides a novel approach to the differential diagnosis in hemoparasite biology.

## Methods

### Preparation of samples

*Plasmodium falciparum* (*Pf*-RBC) was obtained from the Global Resource Bank of Parasitic Protozoa Pathogens (Inha University, Republic of Korea). The glycerol frozen stock of *P. falciparum* field isolate was thawed using a series of sodium chloride solutions in a dropwise manner. *Pf*-RBCs were maintained in 2% leukocyte-free human O^+^ erythrocytes. *Plasmodium falciparum* in vitro cultures were conducted at 5% O_2_, 5% CO_2_, and 90% N_2_ at 37 °C in RPMI 1640 medium (Gibco Life Technologies, USA) supplemented with 25 mM HEPES (Sigma-Aldrich, St. Louis, MO) and l-glutamine, 0.1% 0.1 M hypoxanthine (Sigma-Aldrich), 0.1% gentamicin (Gibco, 10 mg/ml), 0.2% sodium bicarbonate (Sigma-Aldrich), and 10% AlbuMAX I (Gibco). The *P. falciparum *in vitro culture was maintained for three cycles with fresh erythrocytes to obtain a freshly infected ring stage for ODT examination. *Babesia mictori*-infected RBCs (*Bm*-RBCs) were obtained from the patient during the diagnosis and used directly for tomographic diagnosis. The *Bm*- and *Pf*-RBC samples were diluted in phosphate-buffered saline (PBS) solution to approximately 10^6^ RBCs/ml. Ten microliters of the diluted samples was sandwiched between two 25 × 50 mm cover glasses (Matsunami Glass Ind., Ltd., Osaka, Japan).

### Optical diffraction tomography set-up

The Holographic-2H commercial optical diffraction tomography (ODT) system (HT-2H, Tomocube Inc., Republic of Korea) was used to develop and measure *Bm*- and *Pf*-RBCs. The ODT system acquisition was performed using a commercial off-axis Mach-Zehnder interferometric set-up with a digital micromirror device (DMD). A diode-pumped solid-state laser (*λ* = 532 nm) was used for illumination. The beam from the source was split into a sample and referenced by a beam splitter. After beam reflection from the DMD, where the time-multiplexed hologram patterns are projected, the sample beam impinges onto a sample with a controlled illumination angle [20], and the light scattered by the sample is transmitted to the camera by an imaging system consisting of an objective and a tube lens. At the camera plane, the object beam interfered with a plane reference beam on the camera plane, and in-plane off-axis holograms were captured. In total, during this procedure 49 2D inter-reference patterns were recorded to construct a tomogram, and the recording took about 0.1 s. The theoretical spatial resolutions of the imaging system were 110 nm and 360 nm for the lateral and axial direction, respectively [21, 22].

### Generation of 3D RI tomogram images of *Bm*- and *Pf*-RBCs

From the multiple 2D interferograms recorded at the camera, the ODT principle was used to construct a 3D refractive index (RI) through inverse computation of the 3D inhomogeneous Helmholtz equation using a weak scattering or Rytov approximation. Several studies have shown that the Rytov approximation performs excellently in tomographic reconstruction [23, 24]. For each interference map, an optical field image, consisting of both the amplitude and phase information, is retrieved using a field retrieval algorithm. Then, the extracted optical fields are mapped into Fourier space. According to the Fourier diffraction theorem, the inverse Fourier transformed image provides information about the 3D RI distribution of a sample. However, the uncollected side scattering signals caused by the limited numerical apertures of the objective and condenser lenses can lead to poor resolution in the optical axis direction. To fill in the missing information, an iterative regularization algorithm based on a non-negativity constraint was used. The details on the principle and MATLAB code of the ODT algorithm can be found elsewhere [25, 26].

### Quantification of morphological and biochemical parameters of RBCs

Measurements of parasitized *Bm*- and *Pf*-RBCs at the ring stage, referenced to uninfected RBCs, were done using TomoStudio software (Tomocube, Inc.) with specific algorithms to characterize RBCs under study. For each single quantified RBC, 3D distribution of the refractive index (*n* = *x, y, z*) was in the range of 1.34 ≤ *n* ≤ 1.42 and represented hemoglobin (Hb) concentration in the range of 3 ≤ *n* ≤ 37 g/dl. To calculate cytosol volume of RBCs, the sample regions were masked on the RI tomograms so as not to include the background area. In addition, considering inferior resolution in the optical axis direction, the voxels with an RI below the specific value were excluded. To compute Hb concentration of RBCs from tomographic measurements, it was noted that the RI difference between cytoplasm and surrounding medium is linearly proportional to the concentration of the cytoplasm, and the proportionality coefficient, *α*, is called the RI increment (RII) [27, 28]. This relation in our experiments can be written as follows: *n* = *n*_0_ + *αC*, where *n* is an RI in cytoplasmic regions of RBCs, *n*_0_ is the RI of surrounding medium, and *C* is the Hb concentration [27, 28]. The RII value for Hb at the wavelength of 532 nm is 0.149 ml/g [29, 30]. Therefore, the total mass of Hb was calculated by simply integrating Hb concentration over cytosol volume.

### Measurement of membrane fluctuation in RBCs

Membrane fluctuations were initially calculated as dynamic in cell height images at normal-angle laser illumination set perpendicular to the sample. Simply, the 2D RBC interferograms were recorded continuously at a frame rate of 125 Hz for 2.4 s while retrieving a phase delay map Δ*ϕ* (*x*, *y*, *t*) using the field retrieval algorithm. The heights of the 2D RBCs were recorded as *h*(*x*, *y*, *t*). Then, the differences in image heights were calculated as Δ*h*(*x*, *y*, *t*) = Δ*ϕ*(*x*, *y*, *t*) ⋅ *λ*/[(*n*_RBC_) − *n*_*m*_]/2π, where (n_RBC_) is the mean refractive index of RBC cytoplasm and n_m_ the relative index of the surrounding buffer medium. The cell height images obtained were calculated to membrane fluctuation by averaging the root mean square of height difference Σ[Δ*h*(*x*, *y*, *t*) − 〈Δ*h*(*x*, *y*, *t*)〉]^2^/*N*_*frame*_, where *N*_*frame*_ is the total number of frames observed. The calculations and procedures employed were similar to those in previous studies [31]. Fluctuation of live red blood cells is actively driven by thermal and active metabolic energies [32, 33]. It is evident that fluctuation of RBCs is related to parasite pathophysiology [34].

### Statistical analysis

One-way analysis of variance (ANOVA) was applied to determine differences among the three groups and Spearman correlation performed to analyze their correlation. All statistical tests were done using GraphPad Prism (GraphPad Software, San Diego, CA, USA) at *p* ≤ 0.05 level of significance and 95% confidence interval (CI). A total of 86 uninfected RBCs, 32 *Bm*-RBCs, and 65 *Pf*-RBCs were used for analysis in this study.

## Results

### Single cell imaging and morphological differences between ring stages of *B. microti* and *P. falciparum*

**Fig. 1:**
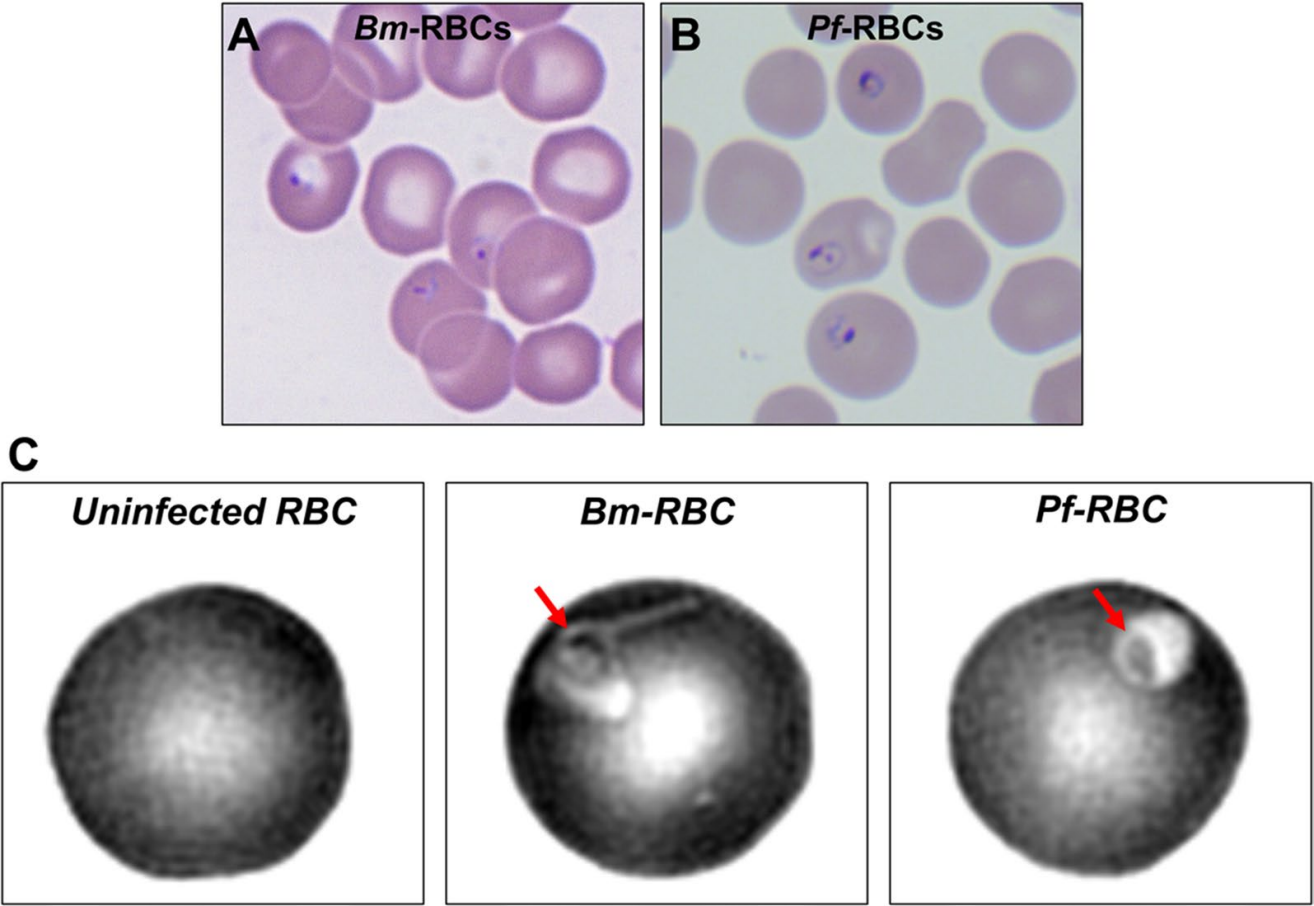
Comparison between *B. microti* and *P. falciparum* diagnoses using conventional microscopy and refractive index tomography. **A** *Babesia microti* detection in patient blood using Giemsa staining. **B** *Plasmodium falciparum* detection in isolate sub-culture using Giemsa staining. **C** Refractive index tomography detection at the ring stage of *B. microti* and *P. falciparum* shown in grayscale with invaded single merozoite (red arrow) and parasitophorous vacuole

**Fig. 2:**
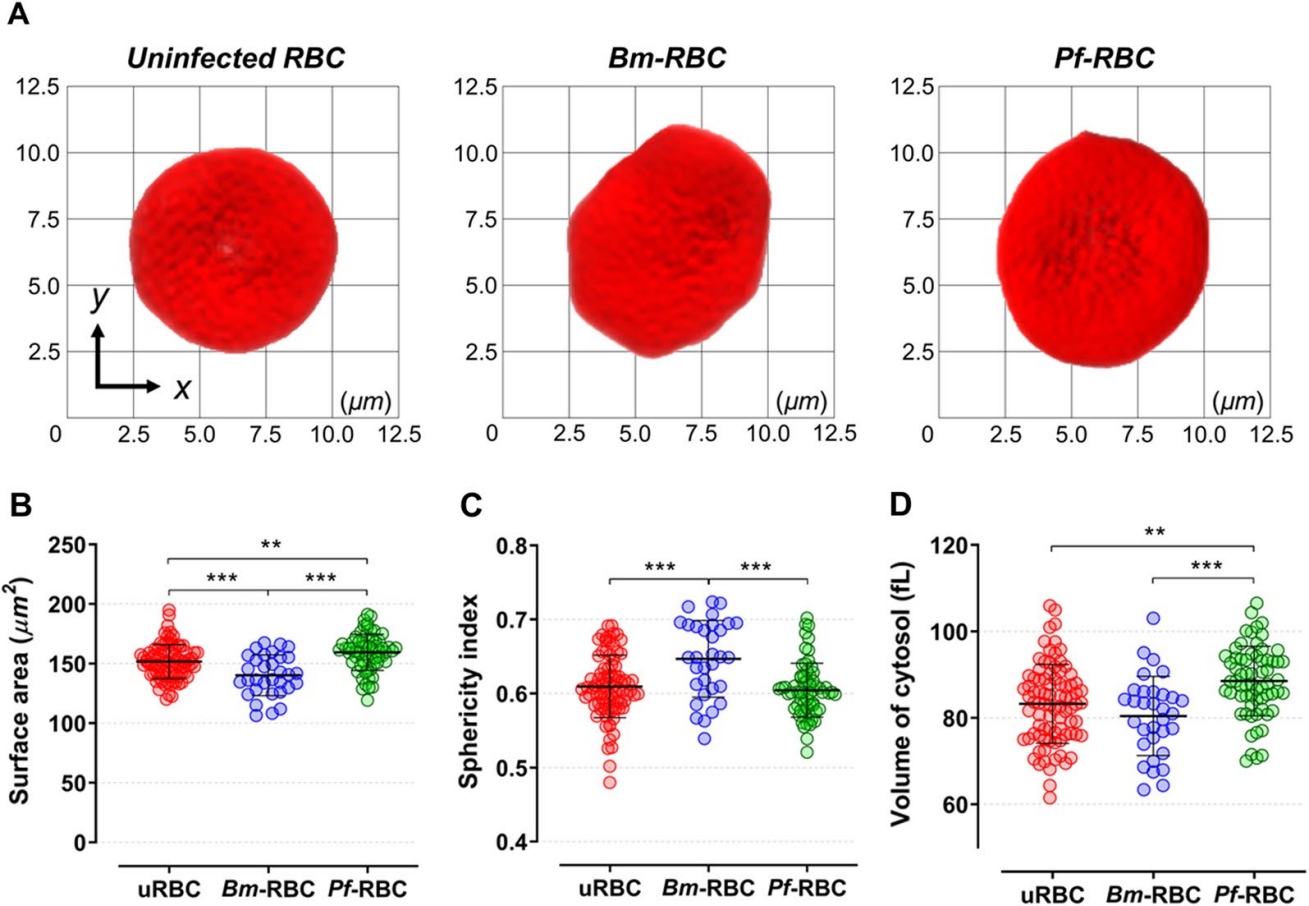
Reconstructive iso-surface and morphological parameter calculation. **A** Reconstructive iso-surface maps are drawn: uninfected RBCs (left), *B. microti*-infected RBCs at the ring stage (*Bm*-RBCs, central), and *Pf*-RBCs (right). The grid background indicates 2.5 µm scale in each square. Morphological parameter calculation in **B** surface area (µm^2^), **C** sphericities, and **C** volume of cytosol (fL). Significant differences are shown as ***P < 0.001 and **P < 0.01

Analysis of *B. microti* and *P. falciparum* field isolates using bright-field microscopy with Giemsa stain presented similar morphological features to those found in this study (Fig. 1A, B). To differentiate the two parasites in an intact live cell, the 3D morphology of healthy and parasitized RBCs was measured using a commercial Holotomography Tomocube instrument (HT-2H). The computational tomography images represented the merozoite position in infected RBCs and parasitophorous vacuoles (Fig. 1C). Based on the tomography images, reconstructive iso-surfaces were generated at *x, y* planes (Fig. 2A). Morphological parameters of uninfected and parasitized RBCs including cellular surface area, sphericities, and volume of cytosol were analyzed from the three measured 3D-RI tomograms. One-way analysis of variance showed significantly smaller size in *Bm*-RBCs and larger size in *Pf*-RBCs compared with uninfected RBCs at the ring stage (Fig. 2B and Table 1). The mean values of RBC sphericities were significantly higher in *Bm*-RBCs; however, uninfected RBCs and *Pf*-RBCs were not significantly different (Fig. 2C and Table 1). The *Pf*-RBCs showed significantly higher cytosol volume than uninfected and *Bm*-RBCs (Fig. 2D and Table 1). Overall, *B. microti* infection resulted in reduced host RBCs with irregular contracted shape, whereas the early ring stage of *Pf*-RBCs showed distended cell size and increased cytosol volume.

**Table 1:** Morphological parameter calculation of uninfected and parasitized RBCs

Variables	Morphological parameters (mean ± SD)		
	Surface area (*µm*^*2*^)	Sphericity index	Volume of cytosol (fL)
uRBCs	151.7 ± 14.3	0.61 ± 0.04	83.23 ± 9.12
*Bm*-RBCs	140.2 ± 17.1	0.66 ± 0.05	80.43 ± 9.16
*Pf*-RBCs	159.0 ± 15.2	0.60 ± 0.04	88.50 ± 8.07

### Single cell imaging and biochemical differences between ring stages of *B. microti* and *P. falciparum*

**Fig. 3:**
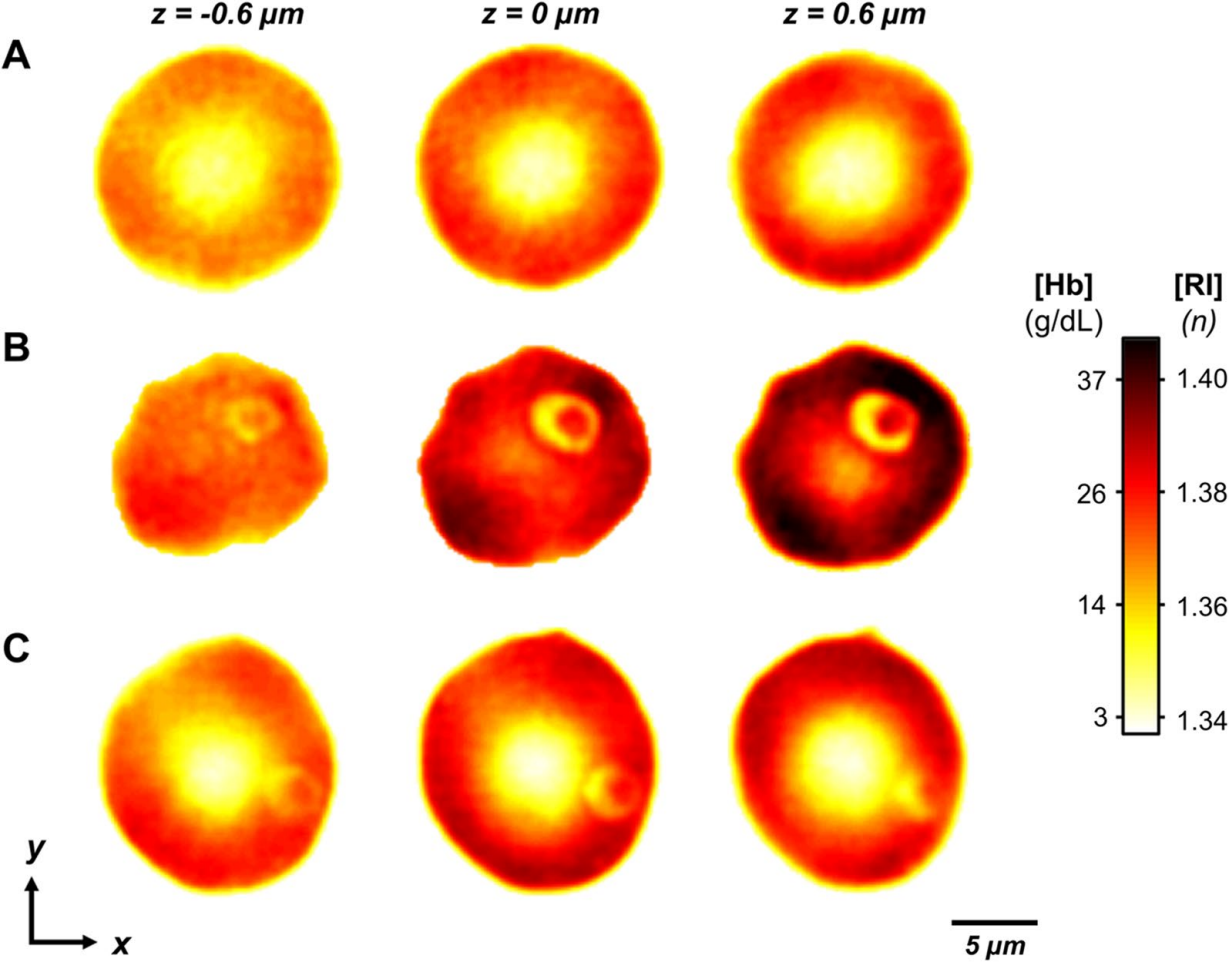
Three-dimensional refractive index maps of uninfected RBCs and ring stage of *B. mictori* and *P. falciparum*. The cross-sectional image shows the middle focus of *x, y* plane ± 0.6 um of **A** uninfected RBCs, **B** *B. microti*- and **C** *P. falciparum*-infected RBCs of section image. The color density map indicates the refractive index (RI, *n*) and the hemoglobin concentration (g/dl)

**Fig. 4:**
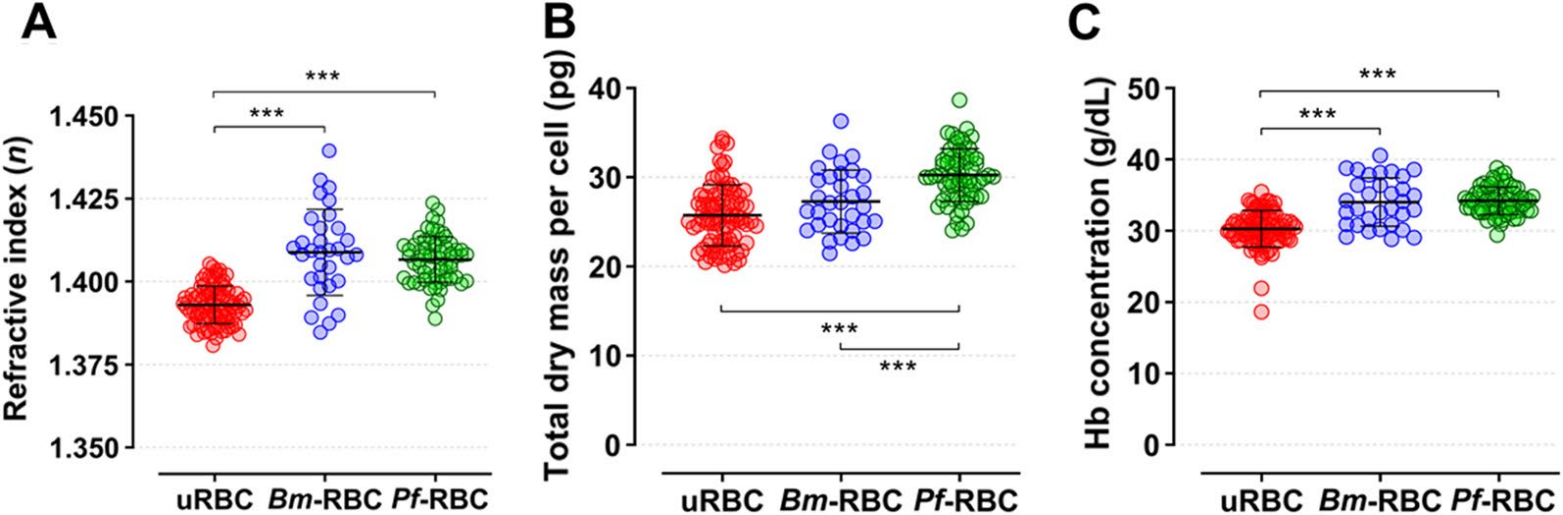
Biochemical parameter measurements of uninfected RBCs (uRBC), *B. microti* (*Bm*-RBC), and *P. falciparum* (*Pf*-RBC) ring stage. **A** Refractive index (*n*), **B** total cell dry mass (pg), and host red cell hemoglobin (Hb) concentration (g/dl). Significant differences are shown as ***P < 0.001

In uninfected RBCs the surface and cross-sectional (Z stack =  ± 0.6 µm) imaging of the refractive index (RI) tomograms clearly showed the biconcave shape and homogeneous distribution of hemoglobin (Fig. 3A). Both *Bm*- and *Pf*-RBCs at early ring stage infection show single merozoite infection (Fig. 3B and C). The parasite-infected RBCs produced parasitophorous vacuoles after the invasion to make an independent environment from host cells, and the region had a low RI value. The overall RI values were significantly elevated in both *B. microti* and *P. falciparum* infection statuses (Fig. 4A and Table 1). The mean values of total dry mass were 25.73 ± 3.43, 27.28 ± 3.52, and 30.25 ± 2.96 pg for uninfected RBCs, *Bm-*RBCs, and *Pf-*RBCs, respectively, which were significantly higher in *P. falciparum* infection (Fig. 4B and Table 1). The mean values of the host red cell hemoglobin concentration were also measured for uninfected RBCs (30.28 ± 2.56 g/dl), *Bm*-RBCs (34.03 ± 3.39 g/dl), and *Pf*-RBCs (34.22 ± 1.95 g/dl) (Fig. 4C Table 1).

### Correlation of parameters for the detailed identification of parasitized cells at ring stage

**Fig. 5:**
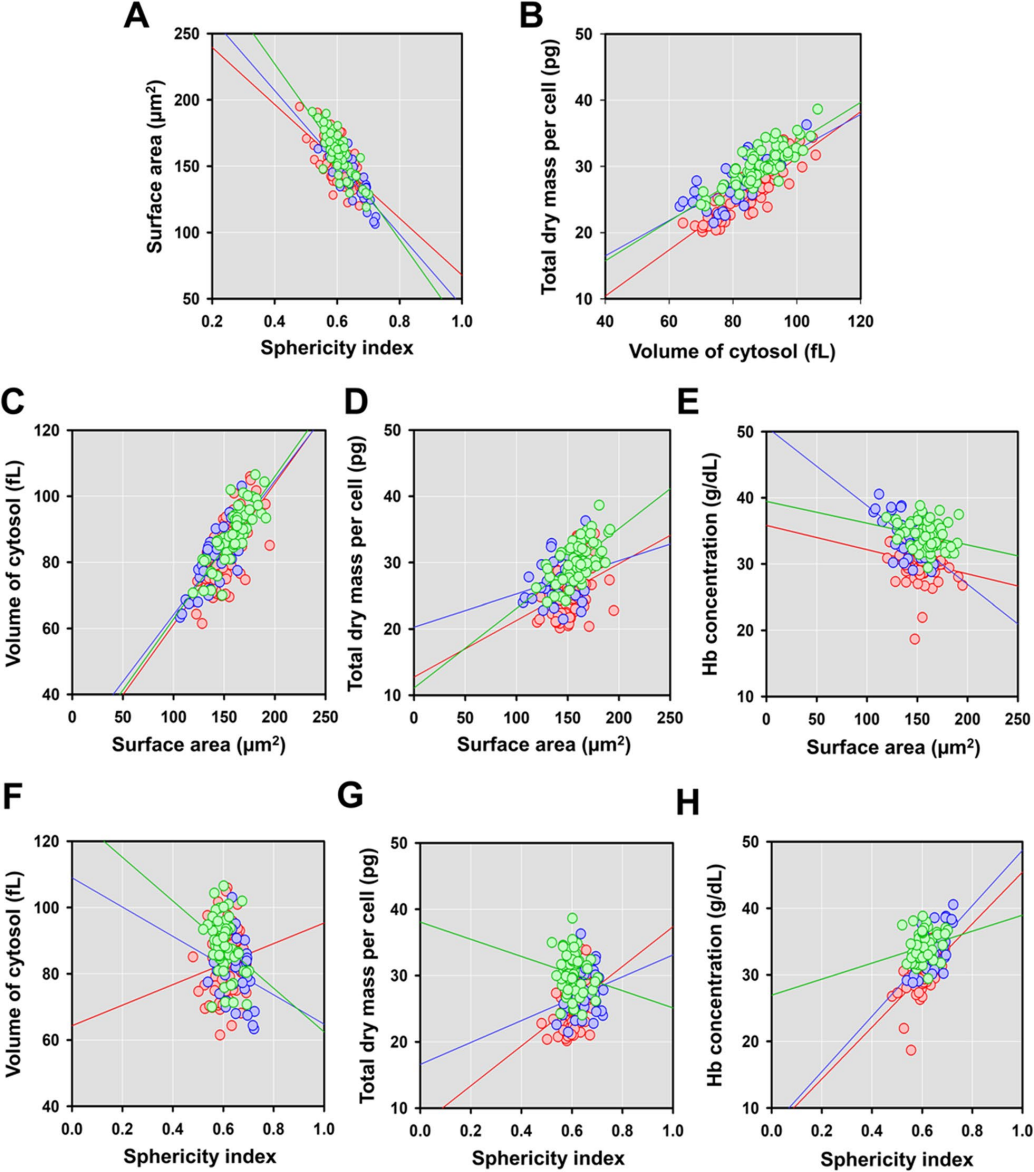
Spearman correlation analysis between the morphological and biochemical parameters for the uRBCs (red), *Bm*-RBCs (blue), and *Pf*-RBCs (green). Correlation analyses between **A** sphericitiy index vs. surface area and **B** volume of cytosol vs. total dry mass per cell. The surface area vs. **C** volume of cytosol, **D** total dry mass per cell, and **E** hemoglobin (Hb) concentration. The sphericity index vs. **F** volume of cytosol, **G** total dry mass per cell, and **H** Hb concentration

To identify detailed differences between *B. microti* and *P. falciparum* ring stage, Spearman correlational analysis was conducted for the parameters assessed (Fig. 5A–H). The correlation value (*ρ*) is shown in Table 2. There were significant positive correlation values in all uninfected RBC, *Bm*-RBC, and *Pf*-RBC volumes of cytosol vs. total dry mass per cell and surface area. Meanwhile, significant negative correlation was observed in sphericities vs. surface area in all of host cell statuses. The remarkable differences between *B. microti* and *P. falciparum* were shown in surface area vs. hemoglobin concentration and total dry mass per cell (Table 2). The sphericities vs. volume of cytosol measurements showed only *P. falciparum* ring stage reached significant negative correlation (Table 2). However, the sphericity vs. Hb concentration indicates *B. microti* had significant positive correlations (Table 2). The sphericity index vs. total dry mass per cell was in contrasting correlation in *Bm*-RBCs and *Pf*-RBCs; however, the values were not significant (Table 2).

**Table 2:** Spearman correlational analysis between tested variables

Variables	Spearman's correlation analysis (*ρ*, *p* value)		
	uRBCs	*Bm*-RBCs	*Pf*-RBCs
Sphericity index vs. surface area	−0.635***	−0.821***	−0.794***
Volume of cytosol vs. total dry mass per cell	0.873***	0.694***	0.818***
Surface area vs. volume of cytosol	0.667***	0.754***	0.811***
Surface area vs. Hb concentration	−0.205	0.599***	−0.257**
Surface area vs. total dry mass per cell	0.357**	0.243	0.618***
Sphericity index vs. volume of cytosol	0.143	−0.249	−0.294*
Sphericity index vs. Hb concentration	0.642***	0.633***	0.225
Sphericity index vs. total dry mass per cell	0.364**	0.242	−0.159

Significant differences are shown as ***P < 0.001; **P < 0.01; *P < 0.05

### Membrane fluctuation between *B. microti*- and *P. falciparum*-infected RBCs

**Fig. 6:**
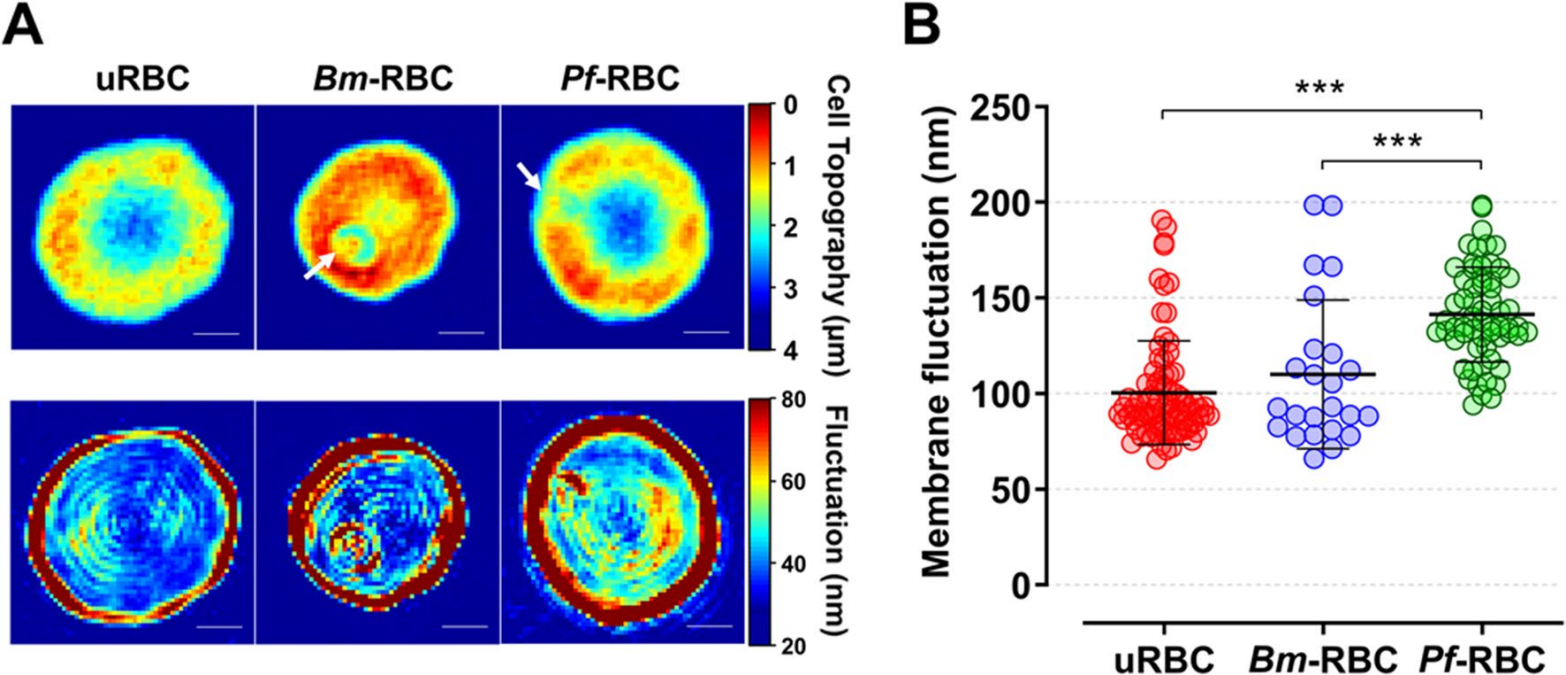
Representative 2D topographical height maps and membrane fluctuation. **A** Two-dimensional topographical images for height and corresponding membrane fluctuation maps of uRBC, *B. microti* (*Bm*-RBC), *P. falciparum* (*Pf*-RBC) infected RBCs from left to right. The arrow indicates a single merozoite in the parasitophorous vacuole. **B** Mean membrane fluctuations of individual RBCs. Significant differences are shown as ***P < 0.001

The target of this study was to investigate fluctuations of RBC membrane after infection with *B. microti* and *P. falciparum*. The red cell topography (µm) and fluctuation height (nm) maps are shown in Fig. 6A. The membrane fluctuation was significantly high in *Pf*-RBC (141.3 ± 24.7 nm) compared to uninfected RBC (100.4 ± 27.1 nm) and *Bm*-RBC cells (110 ± 38.8 nm) (Fig. 6B).

## Discussion

In this study, morphological parameters indicated that the host cell surface area at the ring stage in *B. microti* was significantly smaller than in *P. falciparum*, and sphericity level was higher in *B. microti*-parasitized cells than in *P. falciparum*-infected red blood cells. However, biochemical parameters showed significantly higher levels of hemoglobin in *P. falciparum*-infected red blood cells than in *B. microti*-infected cells, and membrane fluctuations were more active in *P. falciparum*- than in *B. microti*-infected cells.

The conventional microscopic diagnosis for *B. microti* and *P. falciparum* parasitized RBCs is often confusing because of morphological similarity. The ring stage of these parasites is the most similar stage and is frequently misdiagnosed in patients, leading to inappropriate treatments [19]. Thus, in-depth understanding of the properties and differences between the early ring stages of the two parasites is fundamentally important for suitable treatment after accurate diagnosis. To overcome the existing technical limitation, this study applied the optical diffraction tomography (ODT) system to identify detailed characteristics of *B. microti*- and *P. falciparum*-parasitized RBCs using field isolates. The overall morphological parameters including cell surface area and sphericities indicate that *B. microti* infection at the ring stage alters the host red cells to a small globular shape. However, *P. falciparum* infection increases the host cell surface area and cellular cytosol volume. These findings are similar to previous studies [35, 36]. The accurate surface area alteration at the early ring stage measurement was closely related to conventional microscopy diagnosis showing a 0.92-fold decrease in *B. microti* and a 1.05-fold increase in *P. falciparum* infected RBCs. Individual 3D live cell morphological measurements indicated actual differences between *B. microti* and *P. falciparum* ring stage. However, these morphological differences might be difficult to differentiate under conventional microscopic diagnosis because of the blood smear that presents 2D flattened images with stretched RBC shapes.

From the host red blood cell biochemical alteration measurements, *B. microti* and *P. falciparum* infection showed different properties. The biochemical parameters indirectly reflected the metabolic process of the parasites after invasion of host cells [37, 38]. The parasite growth within the host RBCs is known to modify the host cell [39, 40]. Parasites alter infected RBC membrane lipid composition and create new permeation pathways (NPPs), which affect RBC metabolite transportation and membrane cytoskeleton [40–42]. The result shows the increase in host cell hemoglobin concentration in both *P. falciparum* and *B. microti* infections [36]. However, *B. microti* infection induces diminishing cytosol volume, which affects the result of increasing hemoglobin concentration [40]. Thus, the actual increasing of hemoglobin contents of the host cell was elicited by *P. falciparum* rather than *B. microti* infection. Similarly, parasitization by *P. falciparum* induces formation of more parasitophorous vacuoles (PVs) because of maturation of infected RBCs, which subsequently increases cell dry mass [43]. These results clearly show that malaria parasites more actively alter host red blood cells for their replications. These biochemical property differences between *P. falciparum* and *B. microti* infection indicate that *P. falciparum* needs to produce more merozoites than *B. microti*, therefore consuming more cell materials for the nutrient source [36].

Another pathological factor of infected RBCs is a biophysical alteration that is measured by host red cell membrane fluctuation. The host cell membrane fluctuation is closely related to metabolic and thermal energies [32]. Previous studies have shown that membrane fluctuation is higher in *Pf*-RBCs than in normal RBCs [27, 40]. Fluctuation of membrane indicates structural changes which alter its elastic properties between the phospholipid and spectrin network [44]. Intra-erythrocytic parasites induce spectrin-folding transition, which destabilizes the RBC cytoskeleton and subsequently membrane fluctuation [45]. For the same factors, the present study observed that membrane fluctuation is higher in *P. falciparum* compared to *B. microti* infection. This study highlights actual differences between the ring stage of *B. microti* and *P. falciparum*. All the results indicated subtle but significant differences in host cell morphological modifications by the two different parasite infection statuses. Additionally, *P. falciparum* more actively altered host cell biochemical and biophysical properties than *B. microti* infection. This study provides an in-depth understanding of how various hemoparasites utilize the host environment.

## Conclusion

The application of tomography in differential clinical diagnosis of babesiosis and malaria provides a new and easier way to study the pathophysiology of the diseases through clear exploration of the live sample cells using 3D-RI tomograms. This study provides insights for exploring the surface area, volume of cytosol, refractive index, total cell dry mass, and hemoglobin concentration of host RBCs as quantification methods for differential diagnosis of RBCs infected with *B. microti* and *P. falciparum*

## Data Availability

The datasets supporting the conclusions are included in the article.
